# Sub-national variations in general service readiness of primary health care facilities in Ghana: Health policy and equity implications towards the attainment of Universal Health Coverage

**DOI:** 10.1371/journal.pone.0269546

**Published:** 2022-06-03

**Authors:** Martin Ayanore, Robert Asampong, James Akazili, John Koku Awoonor-Williams, Patricia Akweongo

**Affiliations:** 1 School of Public Health, University of Health and Allied Sciences, Ho, Ghana; 2 School of Public Health, Kwame Nkrumah University of Science and Technology, Kumasi, Ghana; 3 Navrongo Health Research Centre, Navrongo, Ghana; 4 Policy Planning Monitoring and Evaluation Division, Ghana Health Service, Accra, Ghana; 5 School of Public Health, University of Ghana, Accra, Ghana; University of Georgia, UNITED STATES

## Abstract

**Background:**

Service availability and readiness are critical for the delivery of quality and essential health care services. In Ghana, there is paucity of literature that describes general service readiness (GSR) of primary health care (PHC) facilities within the national context. This study therefore assessed the GSR of PHC facilities in Ghana to provide evidence to inform heath policy and drive action towards reducing health inequities.

**Methods:**

We analysed data from 140 Service Delivery Points (SDPs) that were part of the Performance Monitoring and Accountability 2020 survey (PMA2020). GSR was computed using the Service Availability and Readiness Assessment (SARA) manual based on four out of five components. Descriptive statistics were computed for both continuous and categorical variables. A multivariable binary logistic regression model was fitted to assess predictors of scoring above the mean GSR. Analyses were performed using Stata version 16.0. Significance level was set at p<0.05.

**Results:**

The average GSR index of SDPs in this study was 83.4%. Specifically, the mean GSR of hospitals was 92.8%, whereas health centres/clinics and CHPS compounds scored 78.0% and 64.3% respectively. The least average scores were observed in the essential medicines and standard precautions for infection prevention categories. We found significant sub-national, urban-rural and facility-related disparities in GSR. Compared to the Greater Accra Region, SDPs in the Eastern, Western, Upper East and Upper West Regions had significantly reduced odds of scoring above the overall GSR. Majority of SDPs with GSR below the average were from rural areas.

**Conclusion:**

Overall, GSR among SDPs is appreciable as compared to other settings. The study highlights the existence of regional, urban-rural and facility-related differences in GSR of SDPs. The reality of health inequities has crucial policy implications which need to be addressed urgently to fast-track progress towards the achievement of the SDGs and UHC targets by 2030.

## Introduction

Access to quality health services is an important function of any country’s health system and a pivot to meet global heath goals. As emphasized by the World Health Organization (WHO), six building blocks define a country’s health system resilience and strength, and enhance the quality of health services provided at country level [1]. At the primary health care (PHC) level, community ownership and engagement, research for development and partnership for health development also form an important part of the six traditional building block for health systems [2,3]. Health service delivery, one of these building blocks relies on the availability, affordability, and acceptability of health services provided in the population [4]. While health service availability and readiness are prerequisites for the delivery of quality and essential health care services, service availability and readiness indicators only remain sufficient to guarantee health systems’ robustness and their ability to deliver quality health care [5]. Globally, health system improvements at the PHC level is a good investment since it facilitates a health system continuum that addresses broad multimorbidity issues and serves as a vehicle for achieving Universal Health Coverage (UHC) [6,7]. Notwithstanding this potential of PHC to deliver UHC targets, evidence is reported as fragmented on how these processes can be achieved [8].

Ghana has a long and enviable history in the area of PHC post-independence (1957). In the early 1990s, Ghana’s Ministry of Health (MoH) action plans included the strengthening of district health management team’s capacity to deliver PHC services through trainings and re-alignment of resources (human and technical) to ensure efficiency in the health sector. The MoH from the year 2000 mapped out strategies to achieve the Government’s vision dubbed Vision 2020 by instituting reforms, leading to the establishment of the Ghana Health Service (GHS) and many other health sector-wide approaches [9]. Other major policy reforms were the introduction of Community-based Health Planning and Services (CHPS) in 2000 as the PHC model, and the National Health Insurance Scheme (NHIS) in 2003. Both the CHPS and NHIS have led to increase in inpatient and outpatient health service coverage [10,11]. The NHIS aim is to guarantee financial risk protection for the vulnerable [12]. These interventions together with other short and medium-term health sector reforms provided a strong anchor to deliver PHC services across the country. Recognizing the essential role of PHC to Ghana’s health service delivery, the MoH in 2016 launched a revised CHPS policy and a revision of the NHIS policy by a Presidential Committee [9]. This was immediately followed by the development of a new CHPS implementation guideline in 2017 [13]. Despite these major milestones in Ghana to advance PHC, broad health system readiness drivers, manifested as inequalities in access to health care [14], geographic inaccessibility of health services [15], vertical and horizontal equity factors [16] are reported in in the country.

Health system readiness can be characterised as one of the attribute measures that define the functionality of the health system or process. Limited studies on health system readiness have been conducted in Ghana. The WHO service availability and readiness assessment (SARA) tool [5] provides a robust approach for country level health teams to track service availability, general service readiness and specific-service readiness across the health system. In this study, general service readiness refers to the overall capacity of health facilities to provide general health services. “Readiness” is defined as the availability of basic amenities, basic equipment, standard precautions for infection prevention, diagnostic capacity and essential medicines to provide those general services. Evidence gathering of such an indicator is thus vital for improving health system functionality and organization in Ghana. Two published studies, Bailey et al [17] and Aboagye et al [18] have examined health system readiness for delivering reproductive health care services while another [19] examined how health workers are system-ready to adapt to new technologies such as e-records. Boyer et al [20] applied a service provision assessment (SPA) data from the National Emergency Obstetric and New-born Care Assessment survey in 2010 to estimate indices of health systems readiness in rural Northern Ghana.

There is no known published study that has applied the WHO SARA survey data to comprehensively assess general service readiness at the PHC level in all geographic regions in Ghana. The WHO SARA tool has the capacity to detect change and progress in country level health system over time, generate evidence for facility and national level decision making and monitor overall health system functionality [5].

We hypothesize that there are sub-national variations in general service readiness in primary health care facilities in Ghana despite country efforts to accelerate progress towards the attainment of UHC goals and a decentralized primary health care system. To accelerate Ghana’s progress towards the attainment of UHC, reduction in inequity and inequalities to health care access and use primarily due to bottlenecks in health system readiness need to be addressed. This study provides national context data for priority-setting and investments required to strengthen health systems in Ghana and across similar context in SSA.

## Methods

### Study design and setting

We used available data from the Performance Monitoring and Accountability 2020 program (PMA2020) survey which were collected in 2017 among women in their reproductive age, households, and service delivery points (SDPs) in Ghana [21]. PMA2017/Ghana Primary Health Care Survey used a two-stage cluster design with urban-rural, major ecological zones as the strata. We assessed general service readiness from the SDPs survey data. With a population of over 30 million, Ghana boasts of a variety of ethnic, linguistic and religious groups. At the time of data collection, Ghana had ten administrative regions but has now been divided into sixteen [22,23]. Data for this study were collected across all ten regions of Ghana.

Health care services are provided by both the public (government-owned) and private (for-profit and not-for-profit) sectors. In descending order, the hierarchy of the public sector and their corresponding facilities are organized as follows: (1) National (teaching hospitals); (2) Regional (regional hospitals); (3) District (district hospitals); (4) Sub-district (health centres and clinics) and (5) Community (Community-based Health Planning and Services (CHPS). Within this hierarchy, Ghana operates a primary health care (PHC) system which focuses on the delivery of essential services at the district, sub-district and community levels. Regional and national levels (regional hospitals and teaching hospitals) focus more on secondary and tertiary health care services although they can provide primary care. They also provide supervisory functions for the lower levels.

Districts do provide secondary health care services but this is often to a reduced extent due to inadequacy of specialised staff. District hospitals typically have at least one qualified medical doctor at post, together with nurses, pharmacists, public health officers, laboratory staff, auxiliary nurses and other support personnel. At the Sub-district level, a health centre or clinic is manned by a resident medical/physician assistant, nurses, midwives, and laboratory staff. The community level (CHPS compound) provides primary care and health promotion to a community or communities (designated as CHPS zones) with a total population size of ≤5000. The CHPS compound, which is the facility, is usually manned by a community health nurse (CHN) or a re-oriented CHN (referred to as Community Health Officer). Although a referral system from a lower level to the next higher level is in place, this system is weak and therefore many individuals visit secondary and tertiary levels for outpatient services.

### Sample size and sampling of Service Delivery Points (SDPs)

A sample of 100 enumeration areas (EA) was drawn by the Ghana Statistical Service from its master sampling frame. In each EA, a census of the public and private health facilities that serve the enumeration area was conducted to populate the list of survey facilities. Since the survey focused on the primary level of care, the district hospital that serves as the referral facility for all the surveyed facilities was purposively selected. A random sample of at most three lower-level facilities SDPs and were selected if they fell within the EA boundaries or if the EA was within the catchment area of the SDP [24]. Facilities of different sizes and levels, from CHPS facilities to health centres and hospitals, were selected to be included in the overall PMA2020 survey sample with the intent to represent the variety of available health facilities in each EA. The final sample included 140 SDPs.

### Data collection

Health care facilities in each enumeration area were surveyed by trained enumerators. The enumerators used structured questionnaire mounted on a mobile-based electronic data collection platform. Heads of facilities were interviewed though face-to-face interviews and data were uploaded into an encrypted and secure cloud server. Data were uploaded as direct responses to the survey tool. Further details regarding the data collection process have been published elsewhere [24].

### Ethical approval

The ethical review boards at the School of Medical Sciences/Komfo Anokye Teaching Hospital Committee on Human Research Publications and Ethics (Kumasi, Ghana; protocol CHRPE/AP/740/1.3), Johns Hopkins University (Baltimore, USA; protocol 7238), and Brigham and Women’s Hospital (Boston, USA; protocol 2016P002284) granted approval to the study [25]. For the purpose of this study, we obtained permission and access to the data for the study from the PMA data repository.

### Data analyses

We assessed general service readiness among SDPs based on the WHO Service Availability and Readiness Assessment (SARA) manual. The SARA guideline describes five domains of general service readiness namely basic amenities, basic equipment, standard precautions for infection prevention, diagnostic capacity and essential medicines [5,26]. Each domain has a number of tracer indicators as shown in Table 1 below.

**Table 1:** SARA domains and tracer indicators for general service readiness.

Domain	Tracer indicators, items or services	Domain score (n = number of tracer items available)
(a) Basic amenities	Power, improved water source, room with privacy, adequate sanitation facilities, communication equipment, access to computer with Internet, emergency transportation	(n/7) × 100
(b) Basic equipment	Adult scale, child scale, thermometer, stethoscope, blood pressure apparatus, light source	(n/6) × 100
(c) Standard precautions for infection prevention	Safe final disposal of sharps, safe final disposal of infectious wastes, appropriate storage of sharps waste, appropriate storage of infectious waste, disinfectant, single-use disposable/auto-disable syringes, soap and running water or alcohol-based hand rub, latex gloves and guidelines for standard precautions	(n/9) × 100
(d) Diagnostic capacity (data not available)	Laboratory tests for: hemoglobin, blood glucose, malaria diagnostic capacity, urine dipstick for protein, urine dipstick for glucose, HIV diagnostic capacity, syphilis RDT and urine pregnancy test	(n/8) × 100
(e) Essential medicines	20 essential medicines: amitriptyline tablet, amlodipine tablet or alternative calcium channel blocker, amoxicillin (syrup/suspension or dispersible tablets AND tablet), ampicillin powder for injection, beclometasone inhaler, ceftriaxone injection, enalapril tablet or alternative ACE inhibitor, fluoxetine tablet, gentamicin injection, glibenclamide tablet, ibuprofen tablet, insulin regular injection, metformin tablet, omeprazole tablet or alternative, oral rehydration solution, paracetamol tablet, salbutamol inhaler, simvastatin tablet or other statin and zinc sulphate (tablet or syrup) Local list on which data were collected (21): Artemisinin combination therapy, Metronidazole, iron supplements, folic acid, Oxytocin, vitamin A, misoprostol, azithromycin, calcium gluconate, magnesium sulfate, ampicillin powder, benzylpenicillin, ceftriaxone, betamethasone/dexamethasone, gentamicin, nifedipine, amoxicillin, oral rehydration salts, zinc, artesunate.	(n/20) × 100
General service readiness index		Mean score of the five domains: (a + b + c + d + e)/5 Since no data on diagnostic capacity were available, mean GSR was calculated as: (a + b + c + e) / 4

The availability of each tracer indicator was coded and captured as “1” if available or “0” if otherwise. The total score for each domain was calculated based on the mean availability of individual items and expressed as a percentage for that domain. We then calculated the mean of all four domain scores and expressed the general service readiness index as a percentage for each SDP. Finally, the overall mean for all 140 SDPs was determined. Next, facilities were then categorized into two; those with lesser general service index and those with greater general service readiness with respect to the mean general service readiness index. We could not factor in the diagnostic capacity domain because no data was not collected on it. Additionally, we assessed essential medicines based on the local list of essential medicines and not necessarily the one provided in the SARA [27].

Descriptive statistics were computed using Stata version 16.0 (Stata MP/StataCorp LLC). Inferential statistics were further done to establish predictive factors for general service readiness. Where expected cell values were less than 5, Fisher’s exact test were computed instead of Pearson’s chi-square test. This was to adjust for the inadequacy of the chi square approximation method by applying the hypergeometric distribution of cell values to test for independence of two categorical variables [28]. Univariate and multivariable binary logistic regression models were fitted to assess the strength of association between dependent variable and explanatory variables. Postestimation test was computed to check model specification and goodness of fit. The Hosmer-Lemeshow goodness-of-fit test (using a grouping of 10) indicated that the model was fit (at p = 0.7064) [a p-value greater than 0.05 validates the fit of the model].

## Results

### Background characteristics

The study comprised a total of 140 SDPs from ten regions in Ghana. The highest number of SDPs (20) were sampled from the Ashanti region whilst the least number of SDPs (7 each) were sampled from the Upper East and Upper West regions. Of all the SDPs, hospitals comprised of 53%, whereas health centres and clinics, and CHPS compounds made up 29% and 18% respectively (**Table** 2). Majority of the facilities (87.1%) are managed by the government, followed by those managed by Faith-based organizations (7.9%). The rest of the facilities are either managed by Non-Governmental Organizations or private individuals under license (**Table** 2). About the same number of facilities were sampled from both urban and rural areas.

**Table 2:** Background characteristics of SDPs.

**Variable**	**Frequency [N = 140]**		**Percentage (%)**	
**Type of facility**				
Hospital	74		52.9	
Health centre and clinics	41		29.3	
CHPS Compounds	25		17.8	
**Managing authority**				
Government	122		87.1	
Faith-based Organization	11		7.9	
Private	6		4.3	
NGO	1		0.7	
**Geographic setting**				
Urban	71		50.7	
Rural	69		49.3	
**Region**	**CHPS Compound (25)**	**Health centres/clinics (41)**	**Hospitals (74)**	**Total**
Ashanti	4	4	12	20
Brong Ahafo	2	5	7	14
Central	4	7	6	17
Eastern	5	3	11	19
Greater Accra	0	5	12	17
Northern	1	4	7	12
Upper East	1	3	3	7
Upper West	1	3	3	7
Volta	1	5	4	10
Western	6	2	9	17

### General health service readiness and variations at the facility level

SDPs in this study were classified into three namely: (1) CHPS compounds, (2) health centres and clinics, and (3) hospitals. The classification is in the order of increasing number of populations served (catchment area). Regarding general health service readiness (GSR) across all three levels of facilities, we found a mean percentage score of 83.4% based on the available data. However, to better understand the context of service readiness, we computed GSR based on the facility type (i.e., CHPS compounds, health centres and clinics, or hospitals). We further categorised SDPs into two based on whether they scored above or below the overall mean percentage score.

Our analysis showed that the mean GSR index was highest among hospitals (92.8%) and lowest among CHPS compounds (64.3%). Among hospitals, only 5.4% had mean GSR below the average GSR for hospitals.

**Fig 1:**
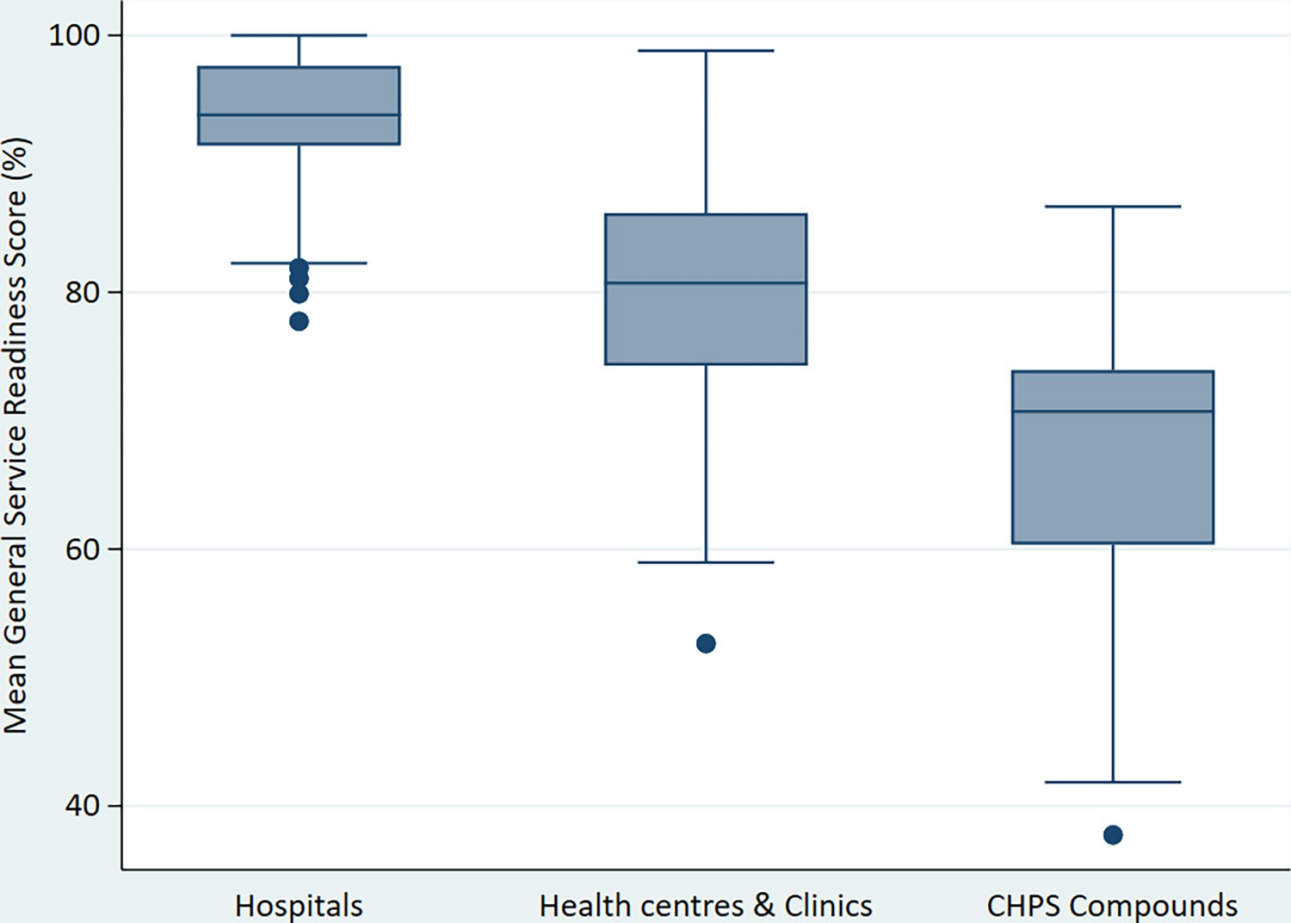
Boxplot of general service readiness score by facility type.

Regarding GSR among health centres and clinics however, more than half (58.5%) had a GSR score below the mean service readiness score across health centres and clinics (78.0%). Furthermore, although the mean GSR at the CHPS level was 64.3%, the vast majority (96.0%) of CHPS compounds scored below it. This is further depicted in the boxplot (**Fig 1**).

### Components of general service readiness

The average score percentages under each component of service readiness indicate the performance and state of each facility under that component. On the whole, hospitals scored the highest in all four components of general service readiness. The highest means percentages scores were in the basic equipment component (97.7%) and standard precautions for infection prevention component (99.3%) (**Table** 3). Health centres and clinics scored higher in the basic equipment and standard precautions for infection prevention components. However, the average score in the essential medicines’ component was much lower (52.6%). We found availability of basic amenities at the health centres and clinics to be fairly satisfactory (70.2%). Among CHPS compounds, basic equipment such as stethoscopes, thermometer, sphygmomanometer (BP apparatus), adult and child scales were usually available. The highest mean score percentage (92.0%) was therefore observed in this component. However, a disproportionately lower mean percentage score, (35.8%), was observed in the essential medicines’ component (**Table** 3). This suggests the possibility of frequent drug shortages or inadequate supply of essential medicines to CHPS compounds. The 56% score in the basic amenities’ component is also expressive of the fact that basic conveniences such as power, improved water source, sanitation and communication equipment are less available in CHPS compounds (**Table** 3).

**Table 3:** Mean percentage score under each component of service readiness by facility levels.

Variable	CHPS (n = 25)	Health centres and clinics (n = 41)	Hospital (n = 74)
**Mean General Service Readiness score** **(Overall mean score = 83.4%)**	64.3%	78.0%	92.8%
Below Mean percentage score	24 (96.0%)	24 (58.5%)	4 (5.4%)
Above Mean percentage score	1 (4.0%)	17 (41.5%)	70 (94.6%)
**Components of Service readiness**			
Basic amenities	56.0%	70.2%	86.8%
Basic equipment	92.0%	98.0%	97.7%
Standard precautions for infection prevention	73.5%	91.2%	99.3%
Essential medicines	35.8%	52.6%	87.5%
Diagnostic capacity*	n/a	n/a	n/a

*Data on Diagnostic capacity were not collected and therefore not included in this analysis.

### Subnational variations in general service readiness

**Fig 2:**
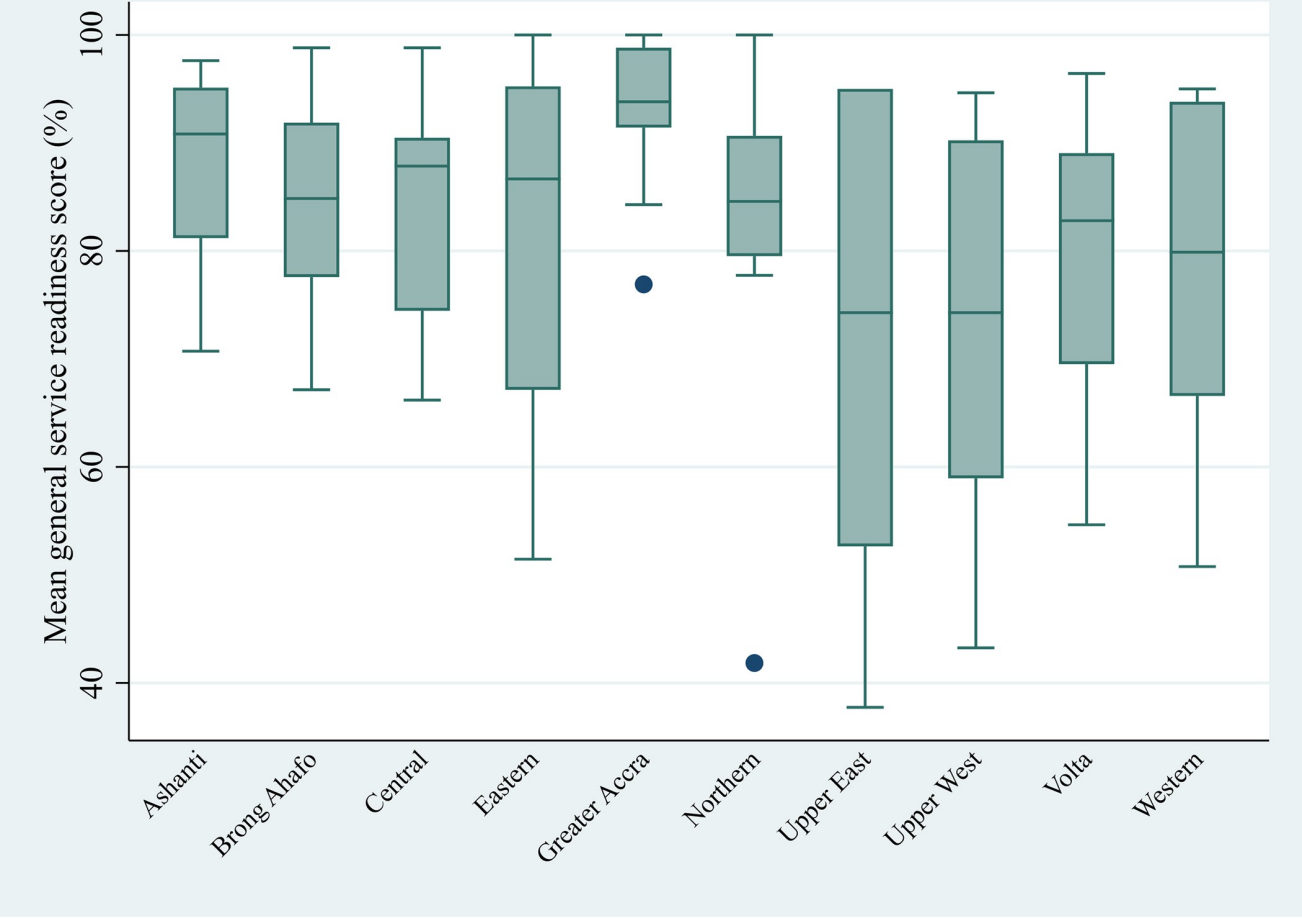
Box plots of general service readiness score by region.

Regarding the regions, a fairly higher percentage of facilities were above the mean GSR across all ten regions. In the Upper West, Upper East and Western Regions, the number of facilities with mean GSR below average were significantly higher as compared to those with higher GSR scores. Interestingly, only 1 (5.8%) of SDPs in the Greater Accra Region had mean GSR below average. In the Volta Region however, analysis show that there are as much SDPs with GSR above average as there are SDPs with GSR below average mean percentage score. **Fig 2** further illustrates the regional differences in GSR. It can be observed that the Volta, Eastern, Western, Upper East and Upper West regions had lower fences of their boxplots below 60%.

### Urban-rural variations in general service readiness

**Fig 3:**
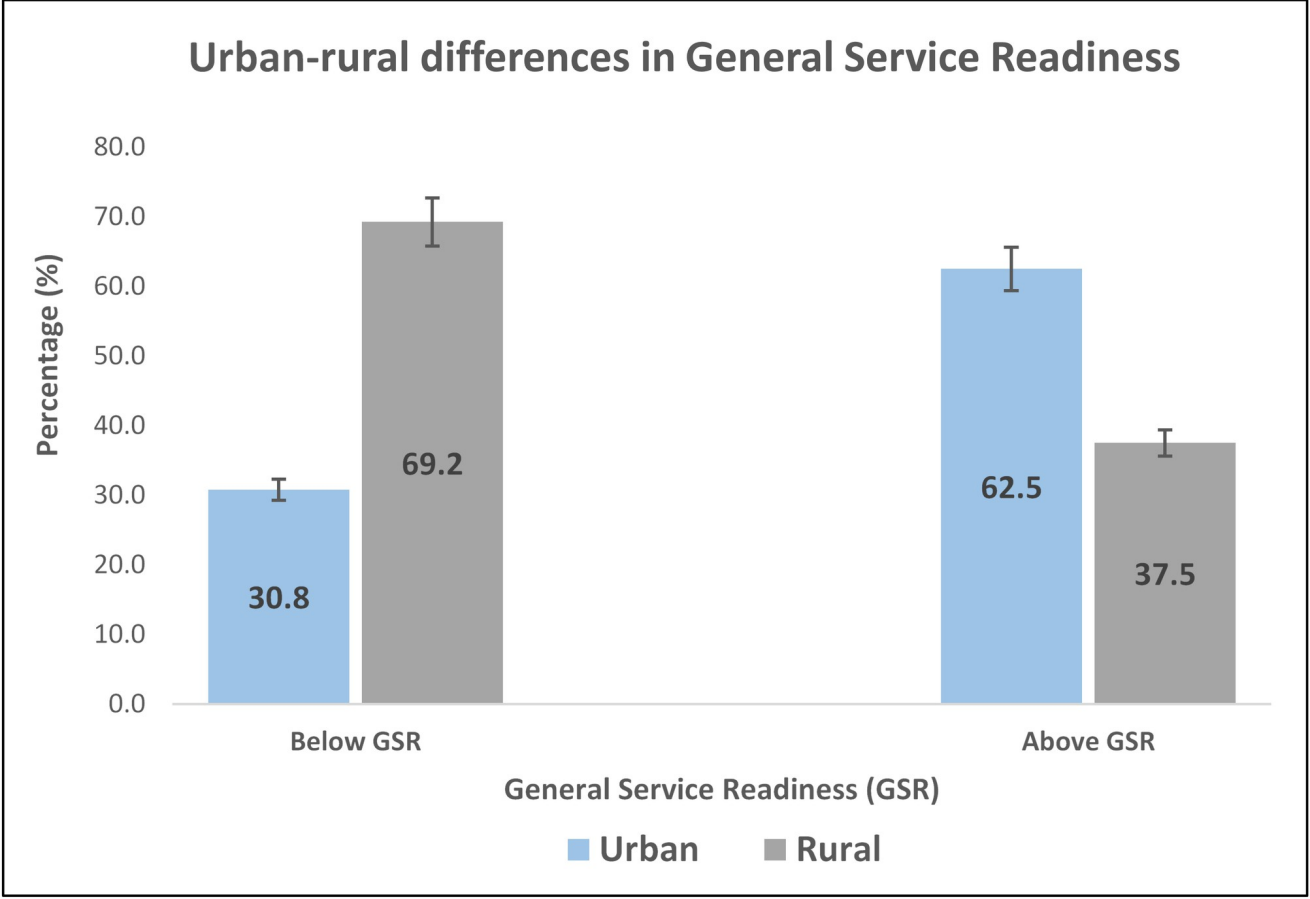
Urban-rural comparison of general service readiness.

A total of 71 SDPs representing 50.7% were sampled from urban areas with the remainder from rural areas. Among facilities with GSR below the mean service readiness score, more than two-thirds were from rural areas (**Fig 3**). In urban areas only, more than three-quarter (77.5%) of SDPs had GSR above the mean service readiness score. However, among SDPs from only rural settings, more than half (52.2%) of SDPs had GSR below the overall mean service readiness score.

### Bivariate and multivariable logistic regression of predictors of general service readiness

Results from the regression analyses are presented in **Table** 4. Bivariate analysis showed that facility type, region, geographical setting (urban/rural), presence of health care workers (HCWs) and receiving formal training in management were statistically significant and associated with general service readiness. We fitted a multivariable logistic regression model after estimating the variance inflation factor of selected predictors. After adjusting for confounders, we found that facility type and region were independently associated with GSR and the results were statistically significant. Compared to hospitals, CHPS compounds, health centres and clinics were less likely to have mean percentage score above the GSR score as expected. We also found that, as compared to the Greater Accra Region of Ghana, health facilities in the Eastern [AOR: 0.01 (0.0003, 0.3932); p = 0.014], Western [AOR: 0.01 (0.0002, 0.3406); p = 0.012], Upper East [AOR: 0.002 (0.00003, 0.1254); p = 0.003], and Upper West Regions [AOR: 0.01 (0.0002, 0.7305); p = 0.035] had very high odds of scoring below the overall GSR.

**Table 4:** Associations between general service readiness and selected facility-related factors.

Variable	Below GSR [N = 52] n (%)	Above GSR [N = 88] n (%)	COR (95% CI)	AOR (95% CI)
**Facility type**				
Hospital	4 (7.6)	70 (79.6)	Ref.	Ref.
Health centres and clinics	24 (46.2)	17 (19.3)	0.04 (0.0124, 0.1322)*	0.0081 (0.0008, 0.0791)*
CHPS compounds	24 (46.2)	1 (1.1)	0.002 (0.0003, 0.0224)*	0.0003 (0.00001, 0.0095)*
**Region**				
Greater Accra	1 (2.0)	16 (18.2)	Ref.	Ref.
Ashanti	5 (9.6)	15 (17.0)	0.19 (0.0196, 1.7962)	0.32 (0.0139, 7.2753)
Brong Ahafo	5 (9.6)	9 (10.2)	0.11 (0.0113, 1.1188)	0.12 (0.0052, 2.5797)
Central	5 (9.6)	12 (13.6)	0.15 (0.0154, 1.4574)	0.78 (0.0408, 15.0419)
Eastern	9 (17.3)	10 (11.4)	0.07 (0.0076, 0.6342)*	0.01 (0.0003, 0.3932)*
Northern	4 (7.7)	8 (9.1)	0.13 (0.0119, 1.3106)	0.06 (0.0026, 1.4471)
Upper East	5 (9.6)	2 (2.3)	0.03 (0.0019, 0.3373)*	0.002 (0.00003, 0.1254)*
Upper West	4 (7.7)	3 (3.4)	0.05 (0.0038, 0.5794)*	0.01 (0.0002, 0.7305)*
Volta	5 (9.6)	5 (5.7)	0.06 (0.0058, 0.6688)*	0.03 (0.0011, 1.0744)
Western	9 (17.3)	8 (9.1)	0.06 (0.0060, 0.5185)*	0.01 (0.0002, 0.3406)*
**Geographical setting**				
Urban	16 (30.8)	55 (62.5)	Ref.	Ref.
Rural	36 (69.2)	33 (37.5)	0.27 (0.1285, 0.5534)*	1.18 (0.2565, 5.4226)
**24-hour presence of HCWs**				
No	14 (26.9)	2 (2.3)	Ref.	Ref.
Yes	38 (73.1)	86 (97.7)	15.84(3.4304,73.1611)*	4.85 (0.4111, 57.1478)
**Formal training in management**				
No	13(25.0)	9 (10.2)	Ref.	Ref.
Yes	39 (75.0)	79 (89.8)	2.93 (1.1516, 7.4339)*	0.72 (0.0916, 5.6372)

_*_ Statistical significance at p-value<0.05 | COR: Crude Odds Ratio | AOR: Adjusted Odds Ratio | CI: Confidence Interval.

## Discussion

The ability and capacity of health facilities to provide consistent services are hinged on the availability of key components of service delivery. In order to ensure the provision of PHC, which is essential and affordable healthcare services for individuals and communities, it is needful to assess SDPs to ascertain their readiness to provide those services. The present study describes the current state of general service readiness (GSR) among selected PHC facilities in Ghana. We further assess potential predictors of GSR of PHC facilities. While the Ministry of Health and Ghana Health Service place much emphasis on the concept of PHC, the present findings show that more need to be done to leverage its potential in contributing to UHC’s health for all agenda.

The overall mean GSR score of all SDPs was 83.4%. Facilities with a general readiness score of 70% are generally considered as having good service readiness [29,30]. Comparing this to the average GSR score in this study the present results show that SDPs in Ghana generally have good service readiness. Our finding affirms the study by Aryeetey et al [31] in which they observed improvement in service readiness post-NHIS in Ghana. Their analysis was however restricted to only mission hospitals in the country. The average score found in this study is relatively higher than those reported in other LMICs such as Nepal, Bangladesh, Haiti, Senegal, Uganda and Kenya, but similar to GSR of hospitals in Malawi, Namibia and Tanzania [32,33]. As Ghana continues to improve the various aspects of health service delivery, it is important to address gaps in other blocks of the health system to sustain the gains made so far. This includes the aspects of health workforce, health financing and leadership and governance.

Among the four components of service readiness assessed in this study, we observed that all facility types had relatively high scores in the basic equipment category. In Ghana, basic equipment such as weighing scales for children and adults, stethoscopes and sphygmomanometers are initially used to assess patients. Having very few or none of instruments such as stethoscopes therefore can be problematic since clinicians sometimes rely on vital signs and other measurements for diagnosis and prescription [34]. However, our analysis found most of the aforementioned equipment are available.

In the standard precautions for infection prevention component, SDPs scored high with the exception of CHPS compounds. This is comparable with the work of Leslie and colleagues [33] in other LMICs. They found that, for lower-level facilities readiness, scores were markedly lower than that of hospitals. This could be due to the fact that lower-level facilities are not mandated to undertake complex medical or surgical operations. As such, some of the requirements for ensuring adequate infection prevention may be loosely adhered to. Protocols for infection prevention are a cardinal indicator of safety in every healthcare setting. Bedoya et al [35] opine that infection prevention and control are a crucial responsibility of a well-functioning health system. Thus, maintaining high standards of preventing accidental or coincidental infections at the health facility demonstrates that the facility is committed to administering effective care for clients. Published literature regarding incidence of healthcare-associated infections further entrench the need to improve infection prevention within SDPs [35,36].

The least performing category we found was the essential medicines component. Intermittent shortage or inadequacy of drugs in pharmacies of health facilities has been a longstanding problem not only in the Ghanaian healthcare system but even in other developed settings [37–40]. Despite state spending on health and the availability of National Health Insurance Scheme, inadequacy of essential medicines prevails in some facilities. The primary reason for this, as reported in the literature, is the inability of the NHIS to reimburse facilities [41–43]. The low readiness scores in the essential medicines component may also be due to increase in the catchment sizes of facilities over time, thereby straining resources available. The downside is that private medicine sellers tend to take opportunistic advantage to gain profits by charging exorbitant amounts from persons with drug prescriptions [43]. We found that although CHPS compounds performed well in other components, a much lower mean percentage score was observed in the essential medicines’ component. This suggests the possibility of frequent drug stock outs or inadequate supply of essential medicines to CHPS compounds as has been found in lower facilities in Nigeria, Vietnam and Bangladesh [27,32,44–47].

Regarding basic amenities, hospitals and health centres/clinics scored higher. The 56% score for CHPS compounds is however indicative of the fact that basic amenities of convenience such as electrical power, improved water source, sanitation and communication equipment are less available. Facilities without electricity may have challenges with vaccine storage whereas those without improved water source may encounter hygiene problems for both health staffs and clients. These findings are almost similar to a study in Uganda [48]. We found that, a number of higher-level facilities had an ambulance or other vehicle available. This is however different from what Ekenna et al [49] reported in Nigeria. In their study, none of the facilities surveyed had a functional ambulance. The recent distribution of ambulances in each constituency in Ghana is therefore a step-up in the resolve to improve service readiness [50].

Among the key findings of this study was the noticeable variation in GSR across regions. More specifically, PHC facilities in the northern part of the country were less likely to score above the overall mean service readiness score. This was also similar to the Eastern and Western Regions where SDPs were less likely to score above the overall mean service readiness score. This indicates regional disparities in general service readiness across the country. The observed differences may be due to the fact that some regions are currently undergoing rapid urbanization which has increased the influx of people and thereby creating the need to expand and improve health services. However, in relatively less developed regions, health care services may not have received the needed attention and therefore left to continue as they are, sometimes with very little improvement. Similar findings of regional disparities in service readiness have been reported in Nigeria and Nepal [32,46].

Moreover, we observed rural-urban disparities in service readiness. More than half of SDPs in rural areas had GSR below the average. This reflects the existence of health inequity in that there is some level of apathy towards the development of rural areas—particularly in the health sector. Our findings corroborate studies elsewhere [33,47,51]. However, the study by Tran et al [44] did not observe any rural-urban differences in GSR. This may be due to differences in sampling methodologies and unavailability of some facility data in their study. The concept of UHC goes beyond the perimeters of urbanised environments to all and sundry to attain that goal.

In the current study, we found that service readiness decreased from the hospital level down to CHPS compounds as expected. CHPS compounds performed the least among all facility types with average readiness score of 64.3%. However, this should not be so since they are much closer to community folk. We argue that CHPS compounds should be more ready to provide general services in order to reduce pressure on higher facilities. The pressure on higher level facilities could be among the reasons why they do not operate to the optimum in terms of secondary and tertiary care. For instance, instead of channelling more resources into secondary and tertiary care services, higher level facilities may be forced to provide more primary care services because more patients skip the lower-level facilities which are essential less ready to provide general services. Although health centres and clinics had an average GSR of 78.0%, this is relatively higher than what was reported in Mongolia [45] and this indicates good service readiness. Variations in service readiness of different facilities have also been reported in Nigeria [46,49].

### Health policy and equity implications

The findings of this study provide a critical lens through which health policies can be reviewed. First, there is the need to take a relook at regional variations in the provision of health care. This is because service readiness is significantly higher for some regions but far below average in others. This is likely due to inequitable distribution of health resources among the regions. As a result, it affects healthcare delivery which can potentially impact on population health outcomes. In order to ensure fairness in national spending on health, we recommend a needs-based approach to guide the disbursement of funds and distribution of healthcare resources to regions [52]. This will help improve general service readiness among PHCs in regions that are lagging behind.

Secondly, we observed significant differences in readiness scores between urban and rural settings. This highlights the need for SDPs in rural areas to be given the needed attention and support so as to improve health service provision and overall population health outcomes. It is common for health resources to be channelled to settings with rapidly increasing population (i.e., urban areas). While this is may be justifiable to some extent, it should not be to the detriment of those living in rural areas. With about 42.7% of the Ghanaian population living in rural areas [53], it is vital to ensure that health facilities and citizens in such areas are not deprived of the needed health resources. There is the need for the intersectoral collaboration between the Ministry of Health and the Ministry of Local Government as well as other ministries to improve overall rural health service readiness and outcomes. We recommend that, in order fast-track progress regarding UHC in Ghana, the Ministry of Health recognises the need to work with all stakeholders including the private sector not only in policy development stage, but more importantly, during policy implementation. This should be done cognisant of the guiding principles of UHC; (1) services delivery, (2) population coverage and (3) financial risk protection.

Moreover, the fact that CHPS compounds performed worse than all the higher-level facilities demonstrate the need to address prevailing gaps in the policy implementation of CHPS. CHPS serve as the first point of call in most communities and it is therefore important for health authorities to ensure that the needed resources—especially basic amenities, equipment and essential medicines are always available. This would not only improve health outcomes but also health-seeking behaviours among community members who cite unavailability of resources as a reason for non-utilization of health services. More importantly, it will help reduce the service delivery burden on higher level facilities.

Finally, our study findings show that in order to accelerate national progress towards health-related SDGs and UHC, service readiness should be among the priority indicators for informing national policy and action. Findings from service readiness and availability surveys can contribute to monitoring progress towards UHC and inform necessary and practical policy actions. Furthermore, stronger intersectoral and multistakeholder collaborations are required to improve general service readiness at the national, regional and local levels.

### Strengths and limitations

Despite the important findings of this study, a number of limitations are worth mentioning. First, the associations presented do not imply causal relationships between service readiness and the said variables. Additionally, no data on diagnostic capacity domain were collected. We acknowledge the likelihood that this may have biased the overall general service readiness. Although crude estimates presented suggest appreciable service readiness, it is important to note that other components were aggregated to compute it. Thus, the comparatively high score may not necessarily be indicative of good performance in each of the domains used to compute the GSR index. More importantly, the data we have analysed has helped identify possible gaps in general service readiness. However, our findings should be interpreted within context. Further research is needed to identify the reasons for the existence of these gaps as these have not been studied. More service availability and readiness surveys using larger samples and data on all five domains of general service readiness are needed.

## Conclusion

This study found that overall, SDPs have good general service readiness score. However, the domains of essential medicines and standard precautions for infection prevention require urgent improvement. Sub-national and urban-rural disparities in readiness are conspicuous from the study findings, which point to varying degrees of health inequity within and between regions. Similarly, service readiness across the different facilities levels showed much variation, with CHPS compounds scoring the least. Although CHPS compounds are at the base of the hierarchy of health facilities, the enhancement of their service readiness is imperative since they are the foremost point of call for most ill individuals within communities. We recommend that regions with general service readiness below average are given much attention and support on a needs-based basis to ensure a collective improvement in service readiness across the country and progress towards Universal Health Coverage.
